# Evaluation of Free Online ADMET Tools for Academic or Small Biotech Environments

**DOI:** 10.3390/molecules28020776

**Published:** 2023-01-12

**Authors:** Júlia Dulsat, Blanca López-Nieto, Roger Estrada-Tejedor, José I. Borrell

**Affiliations:** Grup de Química Farmacèutica, IQS School of Engineering, Universitat Ramon Llull, Via Augusta 390, E-08017 Barcelona, Spain

**Keywords:** absorption, distribution, metabolism, elimination, toxicity, pharmacokinetics, in silico predictions, tyrosine kinase inhibitors, web servers

## Abstract

For a new molecular entity (NME) to become a drug, it is not only essential to have the right biological activity also be safe and efficient, but it is also required to have a favorable pharmacokinetic profile including toxicity (ADMET). Consequently, there is a need to predict, during the early stages of development, the ADMET properties to increase the success rate of compounds reaching the lead optimization process. Since Lipinski’s rule of five, the prediction of pharmacokinetic parameters has evolved towards the current in silico tools based on empirical approaches or molecular modeling. The commercial specialized software for performing such predictions, which is usually costly, is, in many cases, not among the possibilities for research laboratories in academia or at small biotech companies. Nevertheless, in recent years, many free online tools have become available, allowing, more or less accurately, for the prediction of the most relevant pharmacokinetic parameters. This paper studies 18 free web servers capable of predicting ADMET properties and analyzed their advantages and disadvantages, their model-based calculations, and their degree of accuracy by considering the experimental data reported for a set of 24 FDA-approved tyrosine kinase inhibitors (TKIs) as a model of a research project.

## 1. Introduction

In the last decade, about 90% of drug failures were due to poor pharmacokinetic profiles [1,2]: a lack of clinical efficacy (40–50%), unmanaged toxicity (30%), and inadequate drug-like properties (10–15%) [3], but the main problem arises in the late development failure [4]. Faced with this bad prospect, the efforts of the scientific community have focused on improving the drug discovery process by evaluating the ADMET properties in the early stages of drug development [5,6].

Since Lipinski’s rule of five was stated, there has been significant progress in in vitro drug screening characterization technology; however, it is still a time-consuming and expensive process. To overcome this situation, the pharmaceutical industry and research centers have turned their investigation strategy 180 degrees towards in silico predictions based on empirical approaches or molecular modeling [7,8].

Most of the pharmaceutical companies specializing in drug design and development have created their prediction tools, which are not available to the scientific community. On the contrary, there are software companies that have developed ADME prediction tools or databases that can be used under license by research centers or universities at not always affordable prices. So, in general, neither the research laboratories in academia, the small biotech companies, nor the lecturers of Medicinal Chemistry subjects have access to such specialized software. In this sense, some computational research groups have developed various free tools available online that allow for the accurate prediction of most of the relevant pharmacokinetic parameters of molecules of interest.

Recently, Pantaleão et al. carried out an excellent revision of the free webpages available for the prediction of such ADMET properties, analyzing a total of 18 and giving some data about the validation of the predictions [2]. So, taking her job as the starting point and going one step further, we collected and presented a total of 34 available software packages and webservers for predicting ADMET properties. Among this list, 18 websites are free and accessible to the public, which allowed us to statistically evaluate them.

So, our work aimed to study these free platforms and characterize them, taking into account the parameters accessible for prediction, their model-based calculations, their advantages and disadvantages of use, and the confidentiality aspects of the results obtained. Finally, to evaluate their accuracy, 24 tyrosine kinase inhibitors (TKIs) were studied, comparing the results obtained in the prediction with the experimental data.

## 2. Results and Discussion

### 2.1. Pharmacokinetic Profile (ADMET) and Key Pharmacokinetic Parameters

There is no ideal pharmacokinetic behavior that a certain drug candidate should have since it depends on the requirements of the target. For example, an ideal drug planned for occasional pain treatment should have a high absorption for a rapid effect and a fairly fast metabolism to prevent drug accumulation, while a drug planned for chronic disease should ideally have a long elimination halt-time (t_1/2_) to reduce the dose. So, having good pharmacological properties, the drug will achieve the desired biological effect whilst minimizing the adverse effects. Table 1 summarizes the key parameters to understand the pharmacokinetic behavior of a drug that has been considered in our study.

### 2.2. Tools for the Prediction of ADMET Parameters

There are several tools that are available and useful for the in silico prediction of ADMET parameters. On the one hand, there are robust software developed by pharmaceutical or specialized molecular modeling companies, which are available under a paid license. On the other hand, there are free web servers, which are less sophisticated but are fully accessible to academics, and these also offer good in silico predictions.

Some of the software available are external services of specialized companies to which one send the structure of a molecule, and they return the result of the calculations. There is other software available that work under paid license, but they also offer free temporary demos or reduced-price licenses for academic institutions. The main advantage of this type of platform is that you work in-house, which avoids data confidentiality issues. There are also accessible web servers for research and academic environments whose main advantage is that they are free, although some of them require registration with an institutional email. As is common with downloaded software, you run the calculations by yourself, but, in this case, the confidentiality of the data is not always guaranteed.

As stated previously, we have collected information about 34 software and web servers for the prediction of the ADMET parameters. The information about them is presented in Table S1 (see the Supplementary Materials), including the name, a link to the corresponding web page, the authors or distribution companies, the mathematical models used, the input and output format of the data, the accessibility, and other extra information about usability such as computer requirements or data confidentiality. Moreover, the ADMET parameters predicted by each platform are shown in Table S2 (Supplementary Materials). An extra column entitled “Drug-likeness” is included, which corresponds to the fulfillment of Lipinski’s rule of five (MW < 500, log P < 5, hydrogen bond donors <5, and hydrogen bond acceptors <10). As can be easily seen, not all the ADMET predictors can predict all the most important pharmacokinetic properties. Practically, only the software ADMET Predictor from Simulations-Plus covers most of them (https://www.simulations-plus.com/software/admetpredictor/) (accessed on 1 June 2022).

As described in the introduction, the price of commercial software for the prediction of ADMET properties could be a limitation in academia and small biotech companies, particularly if more than one is necessary to cover the different parameters to be predicted. Fortunately, in recent years, free web servers have appeared that allow for the prediction of most of the ADMET properties, thus providing reliable information in the early stages of drug development and allowing one to make the right decisions at the right time. Such free web servers are also an excellent tool for lecturers of Medicinal Chemistry courses because they allow the students to predict such parameters as a part of class exercises. The initial list of 34 tools for the prediction of ADMET properties is reduced to 18 when only free web servers are considered. The resources available and the ADMET properties that they predict are summarized in Table 2.

During the construction of such a list, we have made some observations and experienced a series of drawbacks that we consider worthy of being mentioned:

1. Websites change frequently because they are continuously being improved. Such mutability causes two types of problems: on the one hand, they are not always available to be used, and, on the other hand, the mathematical models can be modified, so the predictions can vary, as Sheridan pointed out in a recent paper [9]. In some cases, the web pages have simply disappeared during the preparation of this manuscript.
2. Some websites, particularly those that allow for uploading a database including several molecules, may take a long time to complete the calculations (in one of the cases tested, 3 h were needed to complete the prediction of 24 compounds).
3. In the final list, it is possible to find specific platforms for a specific pharmacokinetic category, such as MetaTox, NERDD, or XenoSite, which only predict metabolic properties, or DrugLogit, which predicts drug-likeness character. The opposite happens with the ADMETlab, admetSAR, and pkCSM platforms, which predict at least one parameter from each ADMET category.

**Table 2:** Free web servers used for the prediction of ADMET parameters.

Drug-Likeness																		
AMES																		
hERG																		
Carcinogenicity																		
Acute toxicity																		
t_1/2_																		
Cl																		
Sites																		
HLMS																		
Metabolites																		
CYP450																		
PPB																		
BBB																		
Pgp																		
HOB																		
HIA																		
Caco-2																		
pKa																		
log S																		
log P																		
**WEBSERVERS**	ADMETlab [10,11,12]	admetSAR [13]	BioTransformer 3.0 [14,15]	CYP Rules [16]	Drug Logit [17]	FAF-Drugs4 [18,19,20]	Lazar [21]	MetaTox [22]	NERDD [23,24]	OCHEM [25,26]	OSCADD [27]	pKCSM [28]	PreADMET [29,30]	SmartCyp [31,32]	SwissADME [33]	vNN-ADMET [34]	Way2Drug [35]	XenoSite [36]

4. A quick look at Table 2 shows that none of the web servers predict the pKa of a molecule. This is probably one of the most difficult parameters to be found on a free website. In the past years, there are many examples of web pages that were very useful for the prediction of pKa that simply disappeared from one day to another, probably as a result of them being included as a part of commercial software. Recently, the web server MolGpka [37], for the prediction of the pKa of small molecules using a graph-convolutional neural network, has come to help academic researchers, but, for instance, it is not capable of predicting the pKa of an α-carbonyl methylene group.
5. From Table 2, it is also possible to conclude that metabolism is the most predicted category and, within this, the ability of a drug to interact with CYP450 enzymes as a substrate or inhibitor. However, during the early stages of the drug development process, the information related to metabolic stability and the site of metabolism is more relevant for researchers. This type of information is only available on a few specific websites (BioTransformer 3.0, NERDD, and SmartCyp).
6. Just the opposite situation happens with the prediction of the elimination parameters (clearance and half-life time) that are only available on two web servers: ADMETlab and pkCSM.
7. Many websites offer several two-dimensional descriptors (number of heavy atoms, number of rotatable bonds, H-bridges, total polar surface area (TPSA), etc.) that would not be relevant in many cases. Moreover, other websites, especially those that predict toxicity, include long lists of toxicity parameters of difficult interpretation. So, for this research, we have only considered four toxicity parameters: acute toxicity, carcinogenicity, mutagenicity (Ames), and cardiotoxicity (hERG).
8. It is worth mentioning that few web servers predict pharmacodynamic properties. For example, Lazar predicts the lowest observed adverse effect level and the maximum recommended daily dose, which are not considered for this study. In this sense, the main toxicity parameters mentioned in point 7 are included in the study of the pharmacodynamic drug profile.
9. While the use of such websites for the teaching of Medicinal Chemistry is straightforward, the prediction of ADMET properties for compounds under investigation could be problematic due to confidentiality issues. The truth is that only a few platforms include a disclaimer statement saying that the uploaded structures will not be used in any way by the provider of the service. For instance, in the Terms of Use section of SwissADME (provided by the Swiss Institute of Bioinformatics, SIB), it is clearly stated that “SIB is committed to ensuring your privacy and the confidentiality of your personal data”, and a message on the pkCSM website announces that “no molecule information will be retained on the system after being uploaded by the user”.

### 2.3. Comparative Evaluation of the Goodness of the Predictions of the Different Websites

The aforementioned work by Pantaleão et al. [2] covers the descriptions of the different web servers and includes information about the validations carried out with them (in most cases, there are accuracies higher than 70–80%), but no comparative evaluation between them is reported.

**Figure 1:**
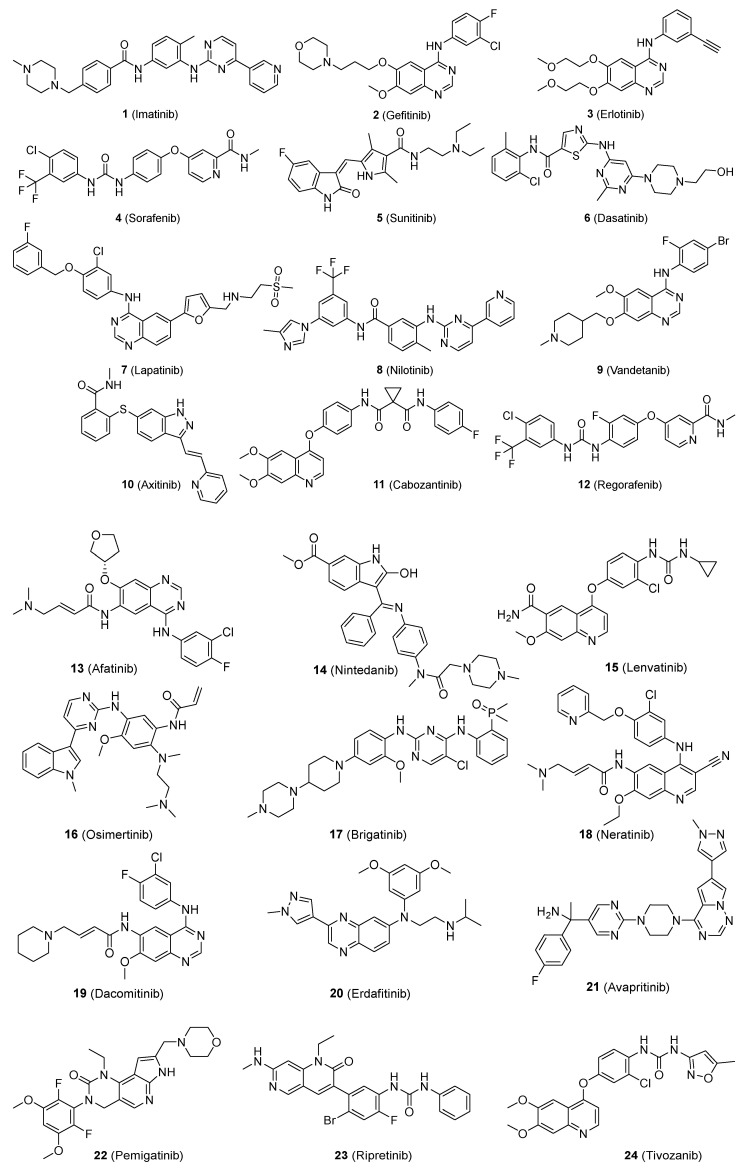
Tyrosine kinase inhibitors included in the database used for the comparative evaluation of the web servers.

To this aim, and following our research in the field of TKIs, we have created a database of 24 FDA-approved TKIs (Table S3 in the Supplementary Materials), which are depicted in Figure 1.

Table S3 includes the entry number, the International Non-proprietary Name (INN) of each compound, the commercial name, the company, the structure, the IUPAC name, the SMILES, the date of FDA approval, the biological targets, the molecular weight, and the experimental pharmacokinetic properties available for such compounds that can be predicted using the websites previously listed. The selection is narrower than that of those databases used for the validation of the different web servers because the mean molecular weight is 487 g/mol, with an SD = 49 g/mol; furthermore, the Shapiro–Wilks test shows a normal distribution (*p*-value = 0.57, α = 0.05). We consider that this is a similar situation to a real research project in which a family of similar compounds could be evaluated using these websites.

To compare the predictions obtained using the different websites and the experimental data, we needed to unify the units of the predictions that, in some cases, are different. Then, the experimental and predicted data were represented by a box-and-whisker or a bar plot, and, finally, a statistical evaluation was performed by a pairwise *t*-test.

It is worth mentioning that, for some compounds, it was not possible to find experimental information for all chosen parameters, as complete pharmacokinetic studies are not always mandatory, so we decided not to consider these molecules in the corresponding comparison. Likewise, the lack of experimental data related to the ability to cross the blood–brain barrier (BBB), and metabolite or metabolic site identification has precluded the inclusion of them as a part of our statistical evaluation of the platforms. Table S4 (Supplementary Materials) contains all the pharmacokinetic parameters predicted for the 24 TKIs using the different web servers (one Excel sheet for each web server). Table S5 (Supplementary Materials) contains the statistical evaluation for each parameter evaluated (one Excel sheet for each parameter).

#### 2.3.1. Erlotinib as a Case Study

**Figure 2:**
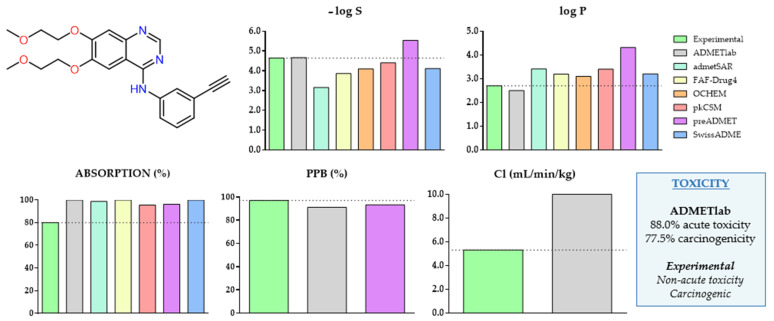
Comparison of the experimental data of Erlotinib (**3**) and the predictions obtained from ADMETlab, admetSAR, FAF-Drug4, OCHEM, pkCSM, preADMET, and SwissADME for solubility (log S), log P, absorption, plasma protein binding, clearance, acute toxicity, and carcinogenicity.

Before starting the comparative evaluation, we used Erlotinib (**3**) as a case study to obtain an initial idea of the goodness of prediction of the free web servers. Solubility (log S), hydrophobicity (log P), oral absorption, plasma protein binding, clearance, and toxicity (in terms of acute toxicity and carcinogenicity) were the parameters on account of which it has been possible to perform this evaluation. The results are summarized in Figure 2.

Erlotinib is a quinazolinamine with a molecular formula of C_22_H_23_N_3_O_4_ and a molecular weight of 393.44 g/mol, and it is barely soluble in water. Erlotinib is an EGFR-TKI approved by the FDA in November of 2004 as a therapy for non-small cell lung cancer (NSCLC) [38].

The solubility and log P predictions show opposite behaviors. Thus, in the first case, the predictions are, in general, lower than the experimental value, while, in the second case, the experimental value is lower than the predicted ones. ADMETlab, OCHEM, and pkCSM gave the predicted values that were closest to the experimental values. On the contrary, admetSAR, and preADMET gave the values that were furthest from the experimental ones. It should be emphasized, as mentioned above, that ADMETlab is the most complete platform in terms of the number of parameters that can be predicted, and it is the only one appearing in all the graphs included in Figure 2. Moreover, we want to highlight its ability to predict the complete set of pharmacokinetic parameters, although, in some cases, the calculations deviate from the experimental values, as is the case for Erlotinib, for the oral absorption, clearance, and acute toxicity.

Finally, we have used MolGpka for the prediction of the pKa values of Erlotinib, with the following results: 5.4 and 5.5 for the protonated nitrogen atoms at the pyrimidine ring of Erlotinib and 13.4 for the ionization of the phenylamino group. Erlotinib hydrochloride has a pKa of 5.42 at 25 °C, so the prediction is really good.

#### 2.3.2. Comparison of Predicted and Experimental Solubilities (log S) for a Group of TKIs

For the evaluation of solubility, expressed as log S (S expressed in mol/L), there are seven websites accessible: ADMETlab, admetSAR, FAF-Drug4, OCHEM, pkCSM, preADMET, and SwissADME. Only 11 of the 24 selected TKIs included experimental values (compounds **1**–**10** and **14**) about solubility, which are included in Table S3.

Water solubility is one of the major properties influencing absorption and, therefore, drug development activities, particularly for projects targeting oral administration. The selected platforms calculate this property as the logarithm of the molar concentration (mol/L). However, SwissADME deserves a special mention, as it offers three prediction models: two topological models (ESOL [39] and Ali et al. [40]) and a third one developed by SILICOS-IT (http://www.silicos-it.be/) (accessed on 1 June 2022).

**Figure 3:**
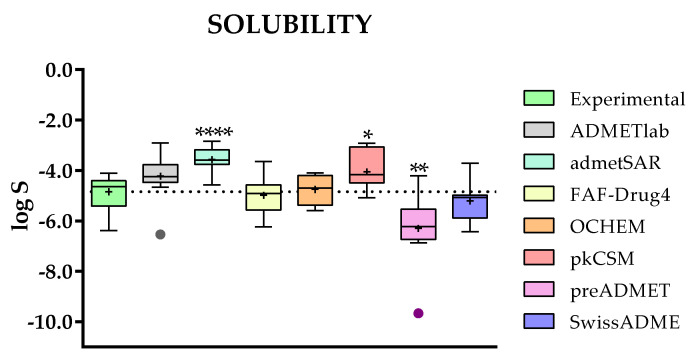
Comparison of the experimental and predicted data for solubility (log S) between ADMETlab, admetSAR, FAF-Drug4, OCHEM, pkCSM, preADMET, and SwissADME. Statistical analysis was performed by a pairwise *t*-test (*n* = 11, * *p* ≤ 0.05, ** *p* ≤ 0.01, *** *p* ≤ 0.001 and **** *p* ≤ 0.0001) versus experimental data.

The statistical analysis between the experimental and predicted log S is presented in Figure 3. The boxplot graphic already shows that some of the web servers produce more accurate predictions than others, which is confirmed by the *p*-values obtained for the pairwise *t*-test. For our group of study (*n* = 11), the best fit is achieved by the FAF-Drug4 and OCHEM platforms, while on the other side, there are admetSAR (*p*-value of 7.56 × 10^−5^), pkCSM, and preADMET, which gave a predicted solubility far from reality.

For solubility, there is a great difference in the accuracy of the prediction evaluated for one compound (Erlotinib) or a group of TKIs. As can be seen in Figure 3, in the case of FAF-Drug4 and OCHEM, the box-plot profiles are almost identical to the experimental data. On the contrary, the ADMETlab platform, which perfectly predicts the solubility of Erlotinib, affords higher predictions than the experimental values for the group of TKIs.

#### 2.3.3. Comparison of the Predicted and Experimental log P for a Group of TKIs

The number of mathematical models that provide log P (log of octanol/water partition coefficient) calculations is huge, so each web page uses a different model for the calculation of this physicochemical property. To mention some of them, FAF-Drug4 uses the XScore package to compute XlogP [41], OCHEM calculates log P with the ALOGPS program [42], and SwissADME returns a consensus log P as the arithmetic average of the values predicted by five methods (XLOGP3 [43], WLOGP [44], MLOGP [45,46], SILICOS-IT, and iLOGP [47]).

**Figure 4:**
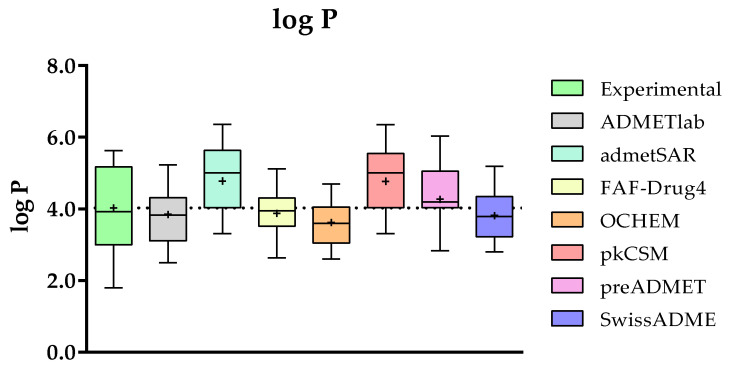
Comparison of the experimental and predicted data for the octanol/water coefficient (log P) between ADMETlab, admetSAR, FAF-Drug4, OCHEM, pkCSM, preADMET, and SwissADME. Statistical analysis was performed by a pairwise *t*-test (*n* = 15) versus experimental data.

In the case of the log P, the experimental data were obtained for a total of 15 compounds (**1**–**9**, **14**–**15**, **17**, **19**, and **23**–**24**) and the corresponding predicted values were obtained from the same seven web servers above. The generated data and the statistical evaluation presented in Figure 4 clearly reveal that all the predictions are close to the experimental values, since none of the websites show significant differences. However, it is important to highlight that the range of the experimental values (1.8 to 5.63) for the group of TKIs is larger than the ranges predicted by each of the websites.

**Figure 5:**
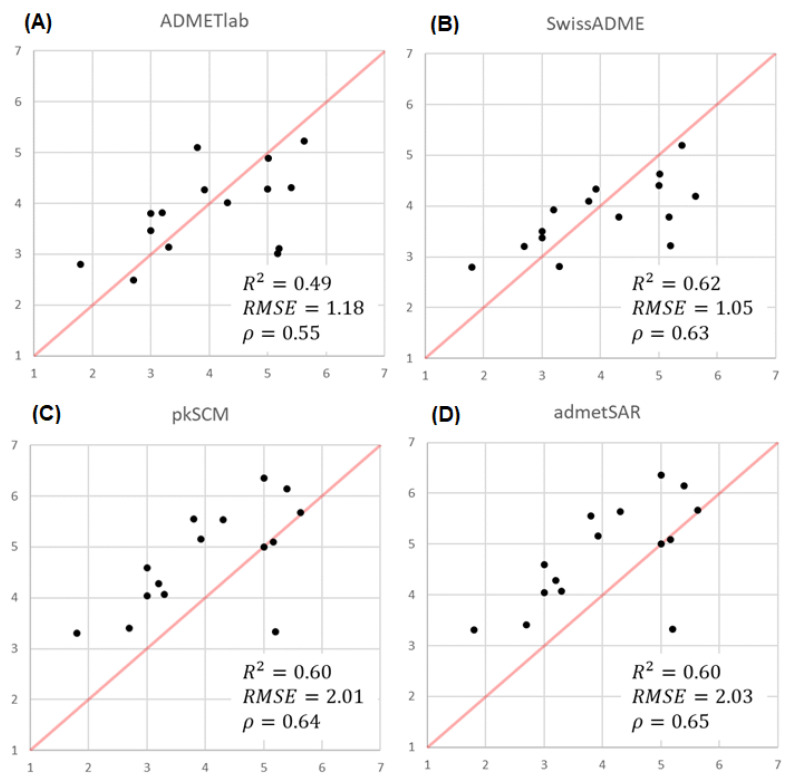
Scatter plot of experimental vs. predicted log P values when using (**A**) ADMETlab, (**B**) SwissADME, (**C**) pkSCM, and (**D**) admetSAR. The correlation coefficient R2, the Root Mean Squared Error, and the Spearman Rank correlation coefficient (\rho) are reported for every regression model.

ADMETlab, pkSCM, SwissADME, and admetSAR are the models with the highest Pearson’s correlation coefficient (Figure 5). From the prediction point of view, the assessment of the regression fitting obtained for these methods shows that ADMETlab and SwissADME offer the lowest Root Mean Square Error (RMSE) value. Particularly, SwissADME is the method with the highest correlation with the experimental values (R2 = 0.62, ρ = 0.63). However, it is important to highlight that the range of the experimental values (1.8 to 5.63) for the group of TKIs is larger than the ranges predicted by each of the websites.

#### 2.3.4. Comparison of Predicted and Experimental Absorption (HIA) for a Group of TKIs

For the study of drug absorption, human intestinal absorption (HIA) is the predicted property offered by the websites ADMETlab, admetSAR, FAF-Drug4, pkCSM, and SwissADME. It is very difficult to predict the absorption of a compound mathematically; therefore, the websites return a classificatory or a probabilistic result. For example, in the case of ADMETlab, a calculated value between 0 and 0.3 means a high probability of having a good absorption, while admetSAR and pkCSM return a probability in terms of percentages. On the contrary, FAF-Drug4 and SwissADME opted for a binary result: high or low absorption.

To compare this property, we have unified data in two categories: high or low absorption, and, thus, the statistical evaluation of the number of compounds in each category has been performed. In this case, the experimental data on the bioavailability of the 24 selected TKIs have been chosen as the control reference. The data have been grouped into two categories (high and low), following the criteria proposed by each of the websites. Thereby, a probability between 0 and 0.3 in ADMETlab and a percentage higher than 30% in pkCSM and over 70% in preADMET and admetSAR mean that a compound has a high human intestinal absorption. Otherwise, the compound is classified into the low group. FAF-Drug4 and SwissADME directly return categorized HIA results.

**Figure 6:**
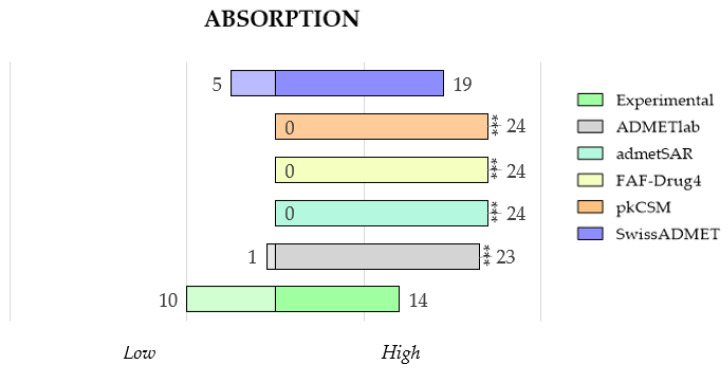
Comparison of experimental and predicted data for human intestinal absorption (HIA), categorized as high and low, between ADMETlab, admetSAR, FAF-Drug4, pkCSM, and SwissADME. Statistical analysis was performed by a pairwise *t*-test (*n* = 24, * *p* ≤ 0.05, ** *p* ≤ 0.01, *** *p* ≤ 0.001, and **** *p* ≤ 0.0001) versus experimental data.

The first thing to highlight is that absorption prediction is not very reliable by any of the platforms, as shown in Figure 6. The first evidence of such lack of precision in the prediction was observed in the Erlotinib case study (Figure 2), where the predicted values were higher than the experimental data. Similar behavior has been observed for the TKIs under study, where most of the predictions indicate a high absorption for all 24 compounds. Only SwissADME can give a low absorption to 5 compounds, although only three of them match their experimental profile.

In conclusion, the results of absorption should be carefully considered and treated together with other parameters before making decisions about real drug candidates.

Although we have selected human intestinal absorption (HIA) for comparison, some of the free websites offer other properties that can give a global idea of the ease of absorption of the drug. Thus, both Caco-2 (human intestinal permeability) and HOB (human oral bioavailability) may be interesting for those compounds designed for oral therapy. Similarly, the PgP parameter (interaction with the P-glycoprotein, a biological barrier that extrudes toxins and xenobiotics) can help in understanding the possible difficulties of drug absorption. Only four web servers, ADMETlab, admetSAR, pkCSM, and preADMET, can predict all the preceding absorption parameters, thus giving a broad vision of the absorption profile of a drug candidate.

#### 2.3.5. Comparison of Predicted and Experimental Plasma Protein Binding (PPB) for a Group of TKIs

**Figure 7:**
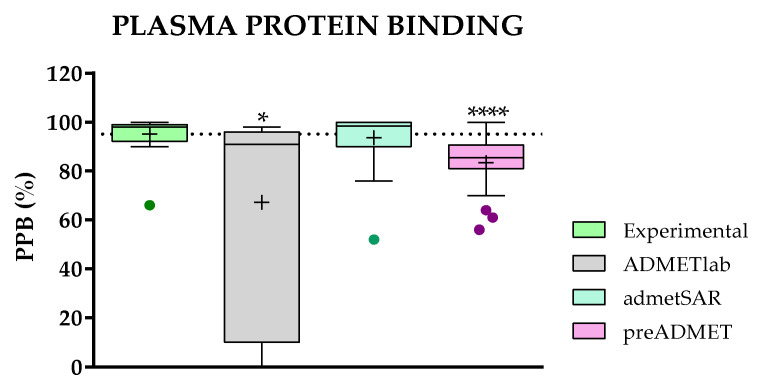
Comparison of experimental and predicted data for plasma protein binding (PPB) between ADMETlab, admetSAR, and preADMET. Statistical analysis was performed by a pairwise *t*-test (*n* = 24, * *p* ≤ 0.05, ** *p* ≤ 0.01, *** *p* ≤ 0.001, and **** *p* ≤ 0.0001) versus experimental data.

Figure 7 summarizes the results of the plasma protein binding (PPB) predictions for the 24 selected TKIs using three websites: ADMETlab, admetSAR, and preADMET.

The experimental data show a tiny variability for such plasma protein binding, meaning that all 24 TKIs tend to be massively bound to plasma proteins. The same behavior is predicted by the admetSAR and preADMET platforms, with a higher variability for this second, supported by a very low *p*-value.

ADMETlab is one of the most interesting websites in terms of the wide range of predicted properties, shows fairly accurate predictions at the same time, and presents a rather abnormal behavior concerning PPB predictions. For 18 of the TKIs considered, ADMETlab perfectly predicts the PPB, but for 6 of them (**13**–**15**, **18**, **22**, and **24**), the predicted values range from 10 to 0, as reflected by a very large box plot.

In studying these six compounds in more detail (their chemical structure, the type of constitutive elements, or their physicochemical properties), and considering the mathematical model used by ADMETlab for the prediction of PPB, no trend has been detected that could explain this abnormality.

For this particular property, admetSAR can be considered the best site, although the range of values is narrow for the TKIs under study.

#### 2.3.6. Comparison of Predicted and Experimental Metabolism for a Group of TKIs

The in silico evaluation of the metabolic profile of a drug candidate by the different web pages mainly consists of the prediction of the probability of such candidate to be a substrate or inhibitor of the five main isoenzymes (1A2, 3A4, 2C9, 2C19, and 2D6) of cytochrome P450 [48,49]. This is essential information, as isoenzymes are key players in drug elimination through metabolic bio-transformations. Therefore, the inhibition of these isoenzymes is certainly one major cause of pharmacokinetic-related drug–drug interactions leading to toxic or other unwanted adverse effects due to the lower clearance and accumulation of the drug or its metabolites [50,51].

**Figure 8:**
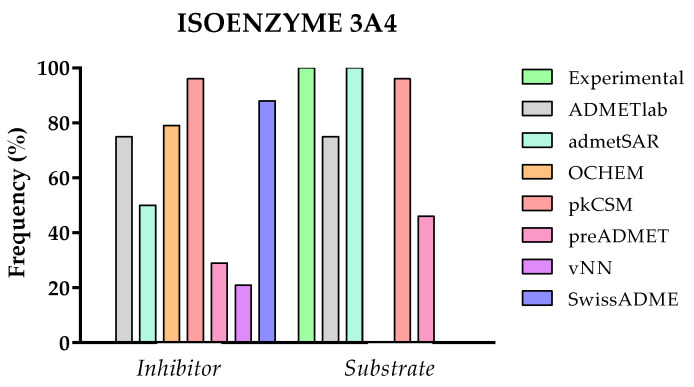
Comparison of experimental and predicted data between ADMETlab, admetSAR, OCHEM, pkCSM, vNN, and SwissADME for the inhibition or being a substrate of the 3A4 isoenzyme (*n* = 24).

Looking at the bibliographic data for the selected 24 TKIs, 3A4 is the isoenzyme through which compounds are mainly metabolized. Thus, to evaluate the prediction accuracy of the platforms ADMETlab, admetSAR, OCHEM, pkCSM, preADMET, vNN, and SwissADME, the effect on the 3A4 isoenzyme has been studied. The data have been standardized and binarily categorized (1 or 0) if they present a probability of acting as a substrate/inhibitor or not. The percentage of compounds predicted to have an inhibitory activity or act as a substrate is represented as a frequency in Figure 8 compared with the experimental data.

Concerning the results depicted in Figure 8, it must be pointed out that OCHEM, vNN, and SwissADME only calculate the probability of being an inhibitor of the 3A4 isoenzyme, not appearing, consequently, in the substrate category. Moreover, vNN only returns a prediction for five compounds (**2**, **3**, **9**, **19**, and **26**), as the rest of the TKIs are out of its applicability domain. This not only happens with this parameter; the same situation has been detected for other properties that it predicts (Pgp, BBB, t_1/2_, hERG, and Ames).

As can be observed in the graph, there is a large variability in the data for both categories evaluated. For example, pkCSM predicts that all 24 TKIs act at the same time as substrates and inhibitors of 3A4 isoenzyme. On the other hand, only 50% are considered inhibitors by admetSAR, and 46% are considered substrates by preADMET. Consequently, it is quite complicated to evaluate and compare the predictive capacity of the different platforms, although the trend in the predictions points out a clear substrate behavior for most TKIs, a result that is consistent with the bibliographic data.

#### 2.3.7. Comparison of Predicted and Experimental Clearance (Cl) for a Group of TKIs

Currently, there are no in vitro laboratory models that allow for the study of drug excretion parameters: clearance and half-life time. Hence, these determinations must be carried out using in vivo assays with animal models that sometimes do not reliably reproduce human physiology. Therefore, until preclinical studies with healthy patients are performed, true information related to such parameters is not available.

Convergently, the elimination parameters are the most difficult to predict mathematically due to the complex biological systems involved. In this sense, only two platforms offer such predictions. On the one hand, ADMETlab predicts both, and, on the other hand, pkCSM only gives information related to clearance.

**Figure 9:**
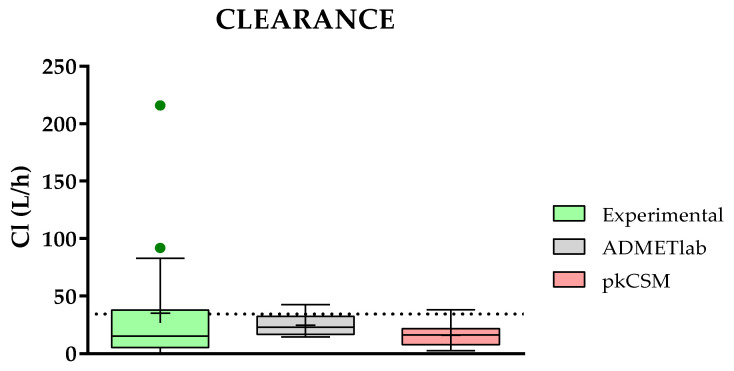
Comparison of experimental and predicted data for clearance (Cl) between ADMETlab and pkCSM. Statistical analysis was performed by a pairwise *t*-test (*n* = 19) versus experimental data.

The prediction of clearance for 19 TKIs (**1**–**5**, **8**, **10**–**11**, **13**–**14**, **16**–**24**) with ADMETlab and pkCSM is shown in Figure 9, together with the corresponding experimental results. As can be noticed, clearance is one of the best-predicted properties, although only two websites can be evaluated. As observed in Figure 8, both ADMETlab and pkCSM demonstrate excellent accuracy in their predictions. However, none of them can predict the two outliers of the experimental data with clearance values around and above 100 L/h.

pkCSM is a platform that predicts almost all the pharmacokinetic parameters described in this work. Although, in some cases, its predictive capability can fail, it is an excellent tool for obtaining an overview of the compounds included in a research project.

#### 2.3.8. Comparison of Predicted and Experimental Toxicity for a Group of TKIs

Besides good biological activity and a favorable pharmacokinetic profile, it is also desirable to design a safe drug with non-existent or at least low toxicity. For our comparison, we selected toxicity parameters including acute toxicity, carcinogenicity, cardiovascular toxicity (hERG), and mutagenicity (Ames). The available experimental values of such toxicity parameters for the 24 TKIs are included in Table S3. An extra column describing other toxicities is included (reproductive, aqueous, or hepatotoxicity).

**Figure 10:**
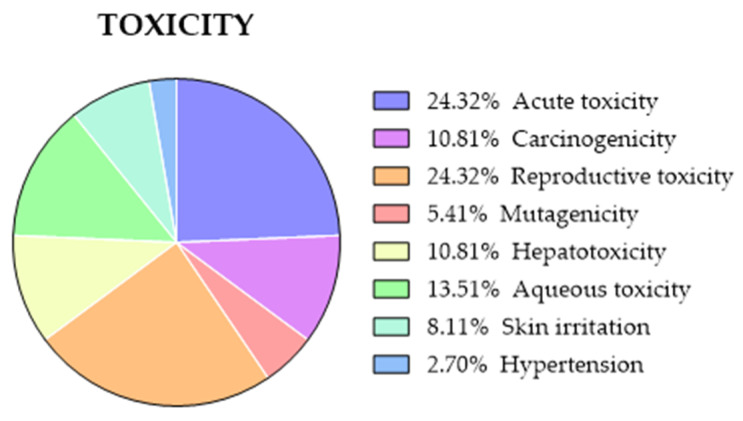
Types of toxicity described for the 24 TKIs under study: acute toxicity (*n* = 9), carcinogenicity (*n* = 4), reproductive toxicity (*n* = 9), mutagenicity (*n* = 2), hepatotoxicity (*n* = 4), aqueous toxicity (*n* = 5), skin irritation (*n* = 3) and hypertension (*n* = 1).

Unfortunately, there is a general lack of experimental data (values or percentages) for some types of toxicity that preclude a statistical comparison with the predicted values. Instead, a ranking has been generated with the most frequent types of toxicity that present the 24 TKIs under study is summarized in Figure 10.

The websites that run toxicity properties are ADMETlab, admetSAR, pkCSM, preADMET, and vNN, although the last one returns an out-of-domain for the 24 TKIs, similar to what happened with metabolism predictions. ADMETlab and admetSAR return the probability of a compound being toxic, while, on the other side, pkCSM and preADMET classify the toxicity of compounds as yes/no or high/medium/low, respectively. All predicted data are included in Table S4.

The predicted values exhibit a large variability and an absence of patterns in all the types of toxicity explored. For cardiac toxicity (hERG), ADMETlab predicts a negative behavior, while preADMET returns a positive toxicity for 22 of the 24 TKIs. In this same sense, the ADMETlab platform predicts a carcinogenic character for one-half of the compounds, while all of them are carcinogenic for admetSAR. For acute toxicity and mutagenicity (Ames), the results are more homogeneous, but the compounds predicted as positive are not the same for the four websites. In conclusion, the variability of the predicted toxicity parameters for a homogeneous group of compounds seems to indicate that such prediction must be considered only as an orientation of the possible toxicity of a drug candidate.

## 3. Materials and Methods

In this paper, we have analyzed 18 web servers that allow for the prediction of ADMET properties at no economic cost. The characteristics of such applications (Table S1, Supplementary Materials) and the pharmacokinetic parameters they predict (Table 2) have been compiled. In addition, a comparative study of the goodness of these predictions has been carried out using the experimental data of 24 FDA-approved TKIs (Table S3, Supplementary Materials) as a model of what a research project of a possible drug candidate would be (narrow distribution of molecular weights close to 500 g/mol). These bibliographic data were obtained from DrugBank (DB) (Wishart research group, University of Alberta, Edmonton, Canada), The Human Metabolome Database (HMDB) (The Metabolomic Innovation Center, Canada), and the Hazardous Substances Data Bank (HSDB) (National Library of Medicine, Washington DC, United States).

The pharmacokinetic predictions for the 24 TKIs were carried out from January to June of 2022, and the data were collected in Table S4 (Supplementary Materials, one Excel sheet for each web server).

To compare the predictions obtained using the different websites and the experimental data, we needed to homogenize their units (as with solubility, log S, and clearance). For other parameters, such as absorption and BBB, it was required to sort the data into binary categories (high/low or yes/no; turn into 1/0 for statistical evaluation) because some websites employed different classificatory levels. Then, the experimental and predicted data were represented by a box-and-whisker plot or a bar graph, and, finally, a statistical evaluation was performed by a pairwise *t*-test carried out by Real Statistics for Excel (C. Zaiontz (2020). Real Statistics Resource Pack software (Release 7.6) (https://www.real-statistics.com/) (accessed on 1 June 2022).

Finally, for some compounds, it was not possible to find experimental information for all chosen parameters, as complete pharmacokinetic studies are not always mandatory, so we decided not to consider these molecules in the corresponding comparison. Each evaluation contains information related to the number of compounds involved. Likewise, the lack of experimental data associated with the ability to cross the blood–brain barrier (BBB) and metabolite identification or sites of metabolism have precluded their integration in our statistical evaluation of the platforms. Of note are the toxicity parameters whose evaluation has been qualitative.

The statistical results of all the comparisons made along with the data tables are included in Table S5 (Supplementary Materials).

## 4. Conclusions

The high number of failures that occur during the development of a drug candidate due to pharmacokinetic problems makes it essential to predict ADMET properties in the initial phases of research. We have analyzed 18 free web servers that are useful for the prediction of physicochemical and pharmacokinetic parameters and are available for academic research laboratories, small biotech companies, and students and lecturers in Medicinal Chemistry. These provide excellent support for research and teaching, making it difficult to justify not using this type of platform.

The conclusions drawn from our study are the following:

1. None of the webservers cover all the physicochemical and pharmacokinetic parameters considered relevant in this study (Table 1). So, in many cases, the simultaneous use of several platforms for the prediction of the required parameters will be needed. ADMETLab is, without a doubt, the one that offers the best coverage of these parameters and the one that offers the best accuracy and precision in the predictions.
2. As mentioned above, the volatility of such free web servers is extremely high, both due to changes in the calculation methodologies and due to the conversion of some of them into commercial software. Each academic or small biotech group interested in using such platforms must carefully select those more stable and more appropriate for their research and monitor the possible changes in the calculation methods.
3. Only a few of the 18 web servers considered guarantee the confidentiality of the structures uploaded for prediction, a condition that is important when developing a new drug candidate. Such a lack of an explicit compromise of confidentiality can set back many academic research groups in the use of these platforms. In our opinion, if only a structure of a molecule is uploaded, it is difficult for it to damage the confidentiality of the project because hundreds are probably uploaded each day; however, if a database containing several similar structures is uploaded, the risk of the unauthorized use of such structures is certainly higher. We strongly suggest that all web servers include a confidentiality statement. If confidentiality is an unnegotiable requirement, two options are available: pkCSM and SwissADME. pkCSM covers a larger range of parameters, at least one of each pharmacokinetic category, but has a lower predictive ability than SwissADME, which is extremely good at covering physicochemical and absorption properties.
4. Concerning the results of such comparisons, octanol/water coefficient and plasma protein binding are the two best-predicted parameters, followed by clearance. On the contrary, solubility and absorption show the worst predicted results due to the complexity of the phenomena involved.
5. Predictions will not avoid the experimental evaluation of candidates during the drug development process, but they can help reduce the number of compounds being studied by offering a global vision of the pharmacokinetic profile of a drug at a low cost and in a short time. More importantly, if a compound is going to fail as a drug, predictions can help in doing it early as possible.

## Figures and Tables

**Table 1:** Key parameters that describe the pharmacokinetic behavior of a drug.

ADMET	Description	Parameters
Physicochemical properties [a]	Intrinsic physical and chemical characteristics of a substance	log P, log S, pKa
Absorption [b]	Transportation of the unmetabolized drug from the site of administration to the body circulation system	Caco-2, HIA, HOB, Pgp
Distribution [c]	Reversible transfer of an unmetabolized drug through the body’s blood and tissues	BBB, PPB
Metabolism [d]	Biotransformation of pharmaceutical substances in the body so that they can be easily eliminated	CYP450, Metabolites, HLMS, Sites
Elimination [e]	The removal of an administered drug from the body	Cl, t_1/2_
Toxicity [f]	The level of damage that a compound can inflict on an organism	Acute toxicity, carcinogenicity, hERG, Ames

[a] Physicochemical properties: log P—log of octanol/water partition coefficient; log S—log of the solubility in mol/L; pKa—negative log of the acid dissociation constant (Ka). [b] Absorption: Caco-2—human intestinal permeability; HIA—Human Intestinal Absorption; HOB—Human Oral Bioavailability; PgP—Permeability glycoprotein. [c] Distribution: BBB—Blood–Brain Barrier penetration; PPB—Plasma Protein Binding. [d] Metabolism: CYP450—Cytochrome P450 inhibition; Metabolites—Metabolite prediction: HLMS—Human Liver Microsomal Stability; Sites—Points susceptible to initiating metabolic transformation. [e] Elimination: Cl—Clearance; t1/2—Half-life time. [f] Toxicity: Acute toxicity—Oral acute toxicity; Carcinogenicity—Capability of inducing cancer; hERG—Cardiac toxicity; AMES—Mutagenicity.

## Data Availability

All the data supporting the reported results and the datasets analyzed or generated during this study and used in this manuscript are publicly available at the repository CORA Repositori de Dades de Recerca (https://dataverse.csuc.cat/, accessed on 1 June 2022) using the following link: https://doi.org/10.34810/data526, accessed on 1 June 2022.

## Supplementary Materials

The following supporting information can be downloaded at: https://www.mdpi.com/article/10.3390/molecules28020776/s1, Table S1: Software and web servers evaluated for ADMET predictions; Table S2: Predicted ADMET parameters for each software and webserver; Table S3: Bibliographic data of 24 FDA-approved TKIs; Table S4: Predictions obtained for 24 TKIs using seven free web servers; Table S5: Comparisons and statistical analysis for each pharmacokinetic parameter.
