# Supplementary material for: Evaluation of Free Online ADMET Tools for Academic or Small Biotech Environments
Source: Molecules. 2023 Jan 12;28(2):776. doi: 10.3390/molecules28020776 (PMC9864198; doi:10.3390/molecules28020776)
Supplement: Supplementary file 1 [file molecules-28-00776-s001.zip › Table S1 Software and webservers evaluated for ADMET predictions.pdf]

NAME	FUNCTION	DISTRIBUTION	PLATFORM	INPUT - single/database	OUTPUT	PREDICTION MODEL	AVAILABILITY	EXTRA INFORMATION
ADMETlab	Accurate and comprehensive predictions of ADMET properties: evaluation & screening	CBDD team of Central South University of Shejiang University	Web-server	Draw, sdf, smiles & txt - s/d	Online & download (csv or pdf)	Multi-task graph attention (MGA) framework	Free webserver	Fast, easy to handle and user friendly
ADMET Predictor	The flagship machine learnig platform for ADMET modeling, with extended capabilities for data analysis, metabolism prediction, and AI-driven drug design	Simulation Plus, Inc.	Software	Mol & sdf - s/d	Data & graph	Finger prints & QSAR models	Possibe DEMO request	Spesialized modules: ADMET modeler, high-throughput pharmacokinetic (HTPK) simulator, MedChem studio, metabolism, biopharmaceutical, toxicity & transporters
admetSAR	admetSAR creates a user-friendly interface to search ADME/T properties	Honhbin Yang (current maintainer), originally created by Feixiong Cheng and Yadi Zhou	Web-server	Smiles - s/d(10)	Online & download (csv)	Support vector machine, k-nearest neighbors & nueral network	Free for non-commetial use	Optimization of lead compounds using scaffold hopping and ADMET screening
ADMEWORKS	System for predicting the pharmacokinetics and safety of drugs and chemicals based on their chemical structure	Fujitsu Global	Software	Mol & sdf - s	Prediction map & descriptors list	Multiple linear regresion	External service	Predictor & model builder modules. Non-disclosure agreement
Bioclipse	Metabolic site predictor of xenobiotic metabolism	Proteochemometric Group, Uppsala University (Sweeden) & Metabolism Team at the European Bioinformatics Institute	Software	Sdf - s/d	Tabular format	Statistical analysis & circular fingerprint	Donation required to download	Provides advanced functionality in fields such as cheminformatics, bioinformatics, semantic web, spectrum analysis, drug discovery, safety assessment & general chemistry education
BioTransformer 3.0	Metabolism prediction webserver	Wishart Research Group, University of Alberta (Canada)	Web-server	Sdf & smiles - s	Online & download (csv or sdf)	Rule-based or maching-learning model	Online or download (it gives all possible transformation - NO ranking)	5 modules: EC-based, CYP450, Phase II, Human Gut Microbial & Environmental Microbial
BIOVIA	Assessment the potential risk posed by unfavorable pharmacokinetic properties and potential toxicity	Dassault Systèmes	Software			JRC QSAR models	External service	European Commission, Joint Research Centre (JRC) (2020): JRC QSAR Model Database. European Commission, Joint Research Centre (JRC)
Computational Resources for Drug Discovery (OSCADD)	Predicting drug-likeness, metabolism & toxicity	Dept. Computational Biology, Indraprastha Institute of Information Technology, India	Web-server	JME editor, mol, sdf & smiles - s	Online or mailed	Machine-learnig (SVM) - QSAR	Java Runtime Environtment required	MetaPred, ToxiPred, DrugMint & QED
CYP Rules	Predicts the metabolizing Cytochromes P450 inhibition	Computational Molecular Design & Detection Laboratory, National Taiwan University	Web-server	Sdf - s	Online	C5.0 algorithm based on Mold2 2D descriptors		
Derek & Meteor Nexus	Expert, knowledge-based software that gives fast and accurate toxicity predictions	Lhasa Limited	Software	Mol & sdf - s/d		QSAR (rule-based & statistical-based)	1 month free demo	Identification, categorization, quantification & control of potentially genotoxic impurities to limit potential carcinogenic risk (ICH M7)
DrugLogit	Clasification as drug or non-drug & disease category	Institute of Chemistry, University of Tartu, Estonia	Web-server	Drug-like properties required - s	Online graph & probability	Logistic regression function	Java Runtime Environtment required	You must enter some information: num. acceptors/donor H, total bonds, PSA, logP, total rings, MW, num. non-H atoms
FAF-Drugs4	In silico ADMET filtering	INSERM, Paris Diderot Un iversity	Web-server	Sdf & smiles - s/d	Download (csv)		The optimized web-server version is available on the Mobyle Portal hosted by the RPBS platform	Possibility to get the results by email
IMPACT-F	Expert system to estimate oral bioavailability of drug-candidates in humans	Pharmainformatic, Germany	Software	Mol & sdf		QSAR models	External service	
IMPACTS	In-silico metabolism prediction by Activated Cytochromes & Transition States (IMPACTS)	Molecular Forecaster Inc.	Software	Draw, mol & sdf - s	Download (csv)	Docking, transition state modelling, and rule-based substrate reactivity prediction	Academic free tool & non-acadmic license	Tutorias available
Lazar	Predictics toxicity datasets by similarity	OpenRiskNet - EU project	Web-server	Draw & smiles - s	Text	Lazy Structure -Activity Relationsip	Log in	Could not find similar substances for threshold 0.2 with experimental data in the training dataset.
MetaTox	Predict the structure, probability & toxicity of metbolites	Institute of Biomedical Chemistry (IBMC), Moscow, Russia	Web-server	Draw & sdf - s	Graph & drawn molecules	Collection of biotransforation reactions & QSAR models	Free webserver	You can select the methabolic reaction
Molecular Discovery	ADME properties	Molecular Discovery Company	Software	Mol & sdf	Draw & values	Grid molecular interactions - NO training set dependent	1, 2 or 3 years contract	MetaSite, MoKa, VolSurf+, MetaDesign

NAME	FUNCTION	DISTRIBUTION	PLATFORM	INPUT - single/database	OUTPUT	PREDICTION MODEL	AVAILABILITY	EXTRA INFORMATION
MultiCASE	Modeling and predicting toxicity of chemicals and xenobiotic metabolites	MultiCASE Inc.	Software	Sdf & smiles - s/d	Document in word, csv, sdf	QSAR models & machine learning algorithms	Licensing or external service	Following ICH M7 methodologies. Ensure securoty for your property information
NERDD	New E-Resources for Drug Discovery - Metabolism	Hamburg University	Web-server	Draw, sdf & smiles - s				Each parameter has to be calculated independently
OCHEM	Online chemical dataset for prediction properties	Helmholtz Zentrum Muenchen (GmbH)	Web-server	Draw, sdf & smiles - s	Download (csv or sdf)	QSAR models & machine learning algorithms	Registration required	
OSIRIS Property Explorer	Toxicity & drug-likeness properties	Idorsia Pharmaceuticals Ltd, Switzerland	Software	Draw	Color bar & percentage	Topological descriptors & fingerprints	Java Runtime Environment and Oracle VirtualBox are required	Predicted information is kept on your computer but for converting smiless to a structure, some text information is sent to servers in Switzerland
Pallas System	Predicts the most common ADME properties (modules)	CompuDrug	Software	Mol & sdf	Table, graph, sdf or RDF		DEMO request - 1 month rental	
Percepta	Platform for *in silico* molecular property calculations	ACD/Labs	Software	Mol & sdf- s/d	Data & color-mapping	QSAR models	DEMO request	
Phoenix	Biosimulation of ADMET properties	Certara	Software	Mol & sdf	Data & graph	Non-compartamental analysis (NCA) & descriptive statistics	External service	
pKCSM	A novel approach to the prediction of pharmacokinetic properties, which relies on graph-based signatures	Bio21 Institute, University of Melbourne	Web-server	Data (smiles) - s/d	Tabular format	Machine-learning platform that relies on distance/pharmacophore patterns encoded as graph-based signatures	Academic free tool & non-acadmic license	Disclaimer: no molecule information will be retained on the system after being uploaded by the user
PK-Sim	Mechanistic modeling of pharmacokinetic & dynamics	Open Systems Phamacology	Software		Graphs	Modular concept to allow efficient multi-scale modelling and simulation	Free download	Software specialized in clinical models, allows to create simulations of existing drugs
PreADMET	Predicting ADMET data & building drug-like library		Web-server	Draw & mol - s	Download (csv, excel, sdf or pdf)		Free webserver	Calculations are run for each of modules (ADME, toxicity & drug-likeness)
QikProp	Rapid ADME predictor for drug candidates	Schrödinger	Software	Mol & sdf		Predictions based on 3D molecular structures; unlike fragment-based approaches	Registration required & trial request	Academic licese
SmartCYP	S ite of metabolism prediction for Cytochrome P450s	University of Copenhagen	Web-server	Draw, mol, sdf & smiles - s	Graph & table	Precomputed activation energies from density functional theory (DFT)	Java Runtime Environment required	
StarDrop	Allows the identification of the region of a molecule that are the most vulnerable to metabolism	Optibrium	Software	Pdb	Data & graph	QSAR models	DEMO request	Every prediction is accompanied by an estimate of confidence
SwissADME	Predictive models for physicochemical properties, pharmacokinetics, druglikeness and medicinal chemistry friendliness.	Molecular Modeling Group of the SIB Swiss Institute of Bioinformatics	Web-server	Draw & smiles - s/d	Online or download (csv & pdf)		Login-free website	Permitted use (private study, internal research and commercial or non-commercial basis. SIB is committed to ensuring your privacy and the confidentiallity of your personal data
vNN-ADMET	Public webserver to predict ADMET properties to build new models based on variable nearest neighbor (vNN)	Telemedicine and Advanced Technology Research Center (TATRC)	Web-server	Draw, smiles & txt - s/d	Categorization table	Variable nearest neighbor (vNN) methodology	Register required	Get the results by email
Way2Drug	Web-server for in silico prediction site of metabolism (SOM)	Dpt. Bioinformatics, Institute of Biometical Chemistry of Rus. Acad. Med. Sci (Moscow)	Web-server	Smile - s	Online & download (sdf & pdf)	Prediction of Activity Spectra for Substrates & LMNA descriptors		
XenoSite	Predicting atomic sites that will undergo metabolized by CYP450	Center for Biological Systems Engineering, Washington University in St. Louis	Web-server	Sdf & smiles - s/d	TSV formal & images (PNG)	Topological & quantum mechanics description in combination with reactivity of atomic sites: neural-network model		
