# Supplementary material for: Evaluation of Free Online ADMET Tools for Academic or Small Biotech Environments
Source: Molecules. 2023 Jan 12;28(2):776. doi: 10.3390/molecules28020776 (PMC9864198; doi:10.3390/molecules28020776)
Supplement: Supplementary file 1 [file molecules-28-00776-s001.zip › Table S2 Predicted ADMET parameters for each software and webserver.pdf]

Software or Webserver	PHYSICOCHEMICAL PROPERTIES			ABSORPTION				DISTRIBUTION		METABOLISM				EXCRETION		TOXICITY				
	log P	*log S*	*pKa*	*Caco-2*	*HIA*	*HOB*	*Pgp*	*BBB*	*PPB*	*CYP450*	*Metabolites*	*HLMS*	*Sites*	*Cl*	*t1/2*	*Acute toxicity*	*Carcinogenic*	*hERG*	*AMES*	*Drug-likeness*
ADMETlab	■	■		■	■	■	■	■	■	■				■	■	■		■	■	■
ADMET Predictor	■	■	■	■		■	■	■	■	■	■	■	■	■	■	■		■	■	
admetSAR		■		■	■	■	■	■	■	■						■		■		
ADMEWORKS	■	■			■		■	■		■							■	■	■	■
Bioclipse										■			■			■		■		
BioTransformer 3.0										■	■		■							
BIOVIA		■			■			■	■	■			■			■		■		
Computational Resources for Drug Discovery (OSCADD)													■			■				■
CYP Rules										■										
Derek & Meteor Nexus											■	■	■			■		■		
DrugLogit																				■
FAF-Drugs4	■	■				■														■
IMPACT-F						■			■											■
IMPACTS										■			■							
Lazar								■								■	■	■		
MetaTox											■									
Molecular Discovery	■	■	■			■		■		■	■		■							
MultiCASE	■	■	■			■		■		■	■		■			■		■		
NERDD										■	■		■							

Software or Webserver	PHYSICOCHEMICAL PROPERTIES			ABSORPTION				DISTRIBUTION		METABOLISM				EXCRETION		TOXICITY				
	log P	*log S*	*pKa*	*Caco-2*	*HIA*	*HOB*	*Pgp*	*BBB*	*PPB*	*CYP450*	*Metabolites*	*HLMS*	*Sites*	*Cl*	*t1/2*	*Acute toxicity*	*Carcinogenic*	*hERG*	*AMES*	*Drug-likeness*
OCHEM	■	■								■										
OSIRIS Property Explorer	■	■														■		■		■
Pallas System	■	■	■	■	■			■			■		■			■	■			■
Percepta	■	■	■	■	■	■	■	■		■			■			■			■	
Phoenix	■	■		■		■	■	■	■					■						
pKCSM	■	■		■	■		■	■		■				■		■			■	
PK-Sim	■	■						■	■	■				■	■					
PreADMET	■	■		■	■		■	■	■	■									■	■
QikProp	■	■		■	■			■	■										■	
SmartCYP										■	■		■							
StarDrop	■	■	■		■		■	■	■	■			■						■	
SwissADME	■	■			■		■	■		■										■
vNN-ADMET							■	■		■		■							■	
Way2Drug													■						■	
XenoSite										■			■							
